# Noninvasive BCL6 Preoperative Screening and Anatomic Patterns of Endometriosis in Patients with Unexplained Infertility

**DOI:** 10.3390/jcm15010377

**Published:** 2026-01-04

**Authors:** Farrah Khoyloo, Camran Nezhat, Zahra Najmi, Quincy Harding, Dahnia Zarroug, Angie Tsuei, Farr Nezhat

**Affiliations:** 1 Camran Nezhat Institute, Center for Minimally Invasive and Robotic Surgery, Woodside, CA 94061, USA; farrah.khoyloo@berkeley.edu (F.K.); zahra.najmi@camrannezhatinstitute.com (Z.N.); quincyadamsharding@gmail.com (Q.H.); dzarroug@stanford.edu (D.Z.); angie.tsuei@camrannezhatinstitute.com (A.T.); 2 School of Public Health, University of California Berkeley, Berkeley, CA 94720, USA; 3 Stanford University Medical Center, Palo Alto, CA 94305, USA; 4 University of California, San Francisco, San Francisco, CA 94143, USA; 5 Computer Science Department, Stanford University, Stanford, CA 94305, USA; 6 Nezhat Surgery for Gynecology/Oncology, Valley Stream, NY 10128, USA; farr@farrnezhatmd.com; 7 Department of OB/GYN, Weil Cornell Medical College of Cornell University, New York, NY 10065, USA; 8 Department of OB/GYN, NYU Long Island School of Medicine, Mineola, NY 11501, USA

**Keywords:** endometriosis, unexplained infertility, precision medicine, endometriosis biomarkers, video laparoscopic surgery, endometriosis surgical techniques, excision of endometriosis, robotic surgery for endometriosis

## Abstract

**Background/Objectives:** Endometriosis is a chronic, inflammatory, estrogen-dependent disease that has historically been underdiagnosed, especially in patients with unexplained infertility. On average, diagnosis is delayed by 11 years, underscoring the need for precision medicine to improve outcomes. To compare disease severity and anatomical distribution of endometriosis between patients with unexplained infertility who underwent noninvasive Receptiva BCL6 testing before surgery and those who did not. **Methods:** A cross-sectional analysis was conducted on 195 women with unexplained infertility and histologically confirmed endometriosis following diagnostic video laparoscopy, with or without robotic assistance. Disease severity was staged using updated guidelines. Anatomical sites of endometriosis were documented. Patients were grouped based on whether they had undergone the Receptiva BCL6 overexpression test prior to surgery. **Results:** Of the 195 patients, 43 underwent Receptiva testing; 41 of them tested positive and were confirmed to have endometriosis during surgery. These patients had fewer affected anatomical regions (3.14 ± 2.09) compared to those without testing (3.93 ± 2.26; *p* = 0.04). The No Receptiva group also had more high-stage cases (70.39% vs. 65.12%, *p*-value: 0.038). In both groups, endometriosis most frequently involved the periureteral region, rectovaginal septum, and ovaries, though ovarian tissue was rarely excised to preserve fertility. **Conclusions:** Among patients with unexplained infertility and confirmed endometriosis, those who had preoperative Receptiva testing showed lower disease burden and severity. These findings support the potential utility of noninvasive testing to enrich diagnostic accuracy and guide earlier, more targeted intervention.

## 1. Introduction

Endometriosis is a chronic, inflammatory, estrogen-dependent, systemic disease characterized by the presence of endometrial-like glands and/or stroma, accompanied by endometrium-like epithelial tissue, involving mostly the reproductive system [1,2]. Many cases also present extragenitally, such as in the bowel or thoracic cavity [3,4,5]. The most common symptoms include pelvic pain and infertility [6]. However, the condition may also be associated with painful intercourse, digestive issues, irritable bowel syndrome, painful and/or heavy menstruation, painful urination, fatigue, depression, among others [6]. However, many patients with endometriosis have also been found to be completely asymptomatic aside from infertility and only became aware of the possibility of diagnosis during infertility evaluation or abdominal and/or pelvic surgeries [7]. Additionally, epidemiological studies have shown that patients with endometriosis are at an elevated risk for other chronic diseases, such as gynecological cancers, cardiovascular diseases, atopic diseases, asthma, and rheumatoid arthritis, among others [8,9,10].

Endometriosis is estimated to affect a range of up to over a billion people worldwide [1]. Studies have found that up to 80% of patients experiencing pelvic pain are diagnosed with endometriosis, while 47% of individuals seeking gynecological care also receive this diagnosis [10,11]. Furthermore, among patients seeking evaluation and diagnosis for endometriosis, those presenting with “unexplained infertility”—defined as individuals unable to conceive despite normal results for ovulation, tubal patency, and semen analysis, as well as undergoing assisted reproductive technologies (ART)—show a prevalence of 90.7% for endometriosis [1].

Historically, the endometriosis patient population has been underserved and overlooked, with many cases going undiagnosed for approximately 11 years. This delay has been attributed to stigmas surrounding reproductive health, barriers to healthcare, and limited access to health education [12,13,14]. As a result, most patients endure not only the physical and mental burdens of endometriosis but also a significant decrease in quality of life [15,16,17,18,19,20,21]. A 2023 meta-analysis, which reviewed twenty-seven studies on the financial costs of endometriosis evaluation and treatment, highlighted the substantial economic burden associated with the disease, including both direct healthcare costs and indirect costs from lost productivity [22].

This underscores the critical need to advance precision medicine for endometriosis, which can help address the significant disparities and clinical challenges in its diagnosis and treatment [23]. Noninvasive biomarkers, such as screening tests for endometrial BCL6 overexpression, have shown promising results in improving the diagnostic accuracy of endometriosis. One study involving a cohort of seventy-five patients reported a remarkable Positive Predictive Value (PPV) of 96% [24]. Other serum biomarkers, including glycoproteins like intercellular adhesion molecule-1 (ICAM-1), are being developed alongside genomic approaches to facilitate the diagnosis of endometriosis and predict its severity [25]. However, despite active genetic studies of endometriosis, there is currently no clear data on genetic markers of the disease suitable for practical clinical use. Researchers are also investigating blood signatures of the disease, such as miRNA sequencing panels [26]. Additionally, oxidative stress markers are being evaluated, with a prominent theory suggesting that an imbalance between reactive oxygen species and antioxidants in the peritoneal cavity contributes to the inflammatory response and the proliferation of endometriotic cells [25]. Furthermore, with the rise in Bio-Tech and Health-Tech innovations in medicine, screening tools such as the Nezhat Free Endometriosis Risk Advisor mobile application have been developed to assist with endometriosis risk evaluation [27]. These advancements are particularly valuable for addressing the underlying issue of unexplained infertility rather than depending on tertiary prevention methods such as oral contraception pills and other treatments.

The use of noninvasive biomarkers, patient history screenings, and pharmaceutical therapeutics can increase suspicion of endometriosis. The ReceptivaDx TM (CiceroDx, Huntington Beach, CA, USA) test detects endometrial BCL6 overexpression in patients who were asymptomatic of endometriosis symptoms other than unexplained infertility or recurring failure in successful conception and implantation [24]. Additionally, it also detects beta-3 integrin expression, which acts as a cellular adhesion molecule that is necessary for successful implantation [24]. BCL6 testing has demonstrated high diagnostic accuracy for endometriosis, with reported sensitivity of 93% and specificity of 96%. While BCL6 overexpression is most commonly associated with endometriosis in women with unexplained infertility, it is a marker of endometrial inflammation and progesterone resistance [24]. However, whether used individually or in combination, none of these tools has advanced to the point of fully replacing diagnostic video-laparoscopy [28,29,30,31]. These noninvasive screening and diagnostic techniques can, however, expedite the decision to perform diagnostic video-laparoscopy, ultimately reducing delays in obtaining a diagnosis and enabling earlier treatment before disease severity worsens and the number of endometriotic lesions increases.

The purpose of this study is to characterize differences in disease severity and anatomical distribution of endometriosis between patients with unexplained infertility who had undergone noninvasive diagnostic testing (Receptiva BCL6 overexpression test) prior to surgical intervention, and those who had not, reflecting current variations in clinical practice.

## 2. Materials and Methods

This retrospective cross-sectional study used data collected from September 2019 to March 2023. All procedures were performed by the senior lead surgeon and his team of clinical fellows at the Camran Nezhat Minimally Invasive Surgery Center, which specializes in endometriosis diagnosis and treatment. The study received approval from the IRB, the Institutional Review Board of the West Coast IRB committee. This study used a convenience sample of all eligible patients who met the inclusion criteria during the study period.

Data collection encompassed patients who had experienced infertility and had undergone one or more unsuccessful infertility treatments, including assisted reproductive technology (ART), hormonal medications, intrauterine insemination (IUI), and/or in vitro fertilization (IVF). After exhausting all conventional infertility treatments, these patients sought evaluation for endometriosis at a center specializing in minimally invasive gynecological surgery. The majority were referred by their reproductive endocrinologist, who had been overseeing their infertility treatment [31].A smaller subset of patients was offered noninvasive biomarker testing for the overexpression of endometrial BCL6 by the referring physician, as a predictive measure of diagnostic accuracy prior to being referred for diagnostic video-laparoscopy [12].

All participants provided informed consent for diagnostic operative video-laparoscopy, with or without robotic assistance, in conjunction with video-hysteroscopy. The procedure included an initial evaluation of the abdominal, pelvic, and uterine cavities, along with an assessment of tubal patency. The classification of endometriosis severity in this study was based on a recently updated staging system [32]. This revised method categorizes endometriosis and adenomyosis into genital and extragenital stages I–IV, corresponding to minimal, mild, moderate, and severe disease, respectively. The staging accounts for both the number and size of endometriotic lesions and their anatomic distribution, including genital and extragenital sites such as the bowel and appendix, providing a more comprehensive assessment of disease extent and severity. When both genital and extragenital staging were documented, the more severe stage was recorded; when only one was available, that value was used [33]. Different biopsies were taken from identified anatomical regions and sent to Stanford Medical Center pathologists were involved for confirmation of the presence of endometriosis.

Patient medical data were securely stored in an electronic health record system (Practice Fusion, San Francisco, CA, USA) throughout the data collection process. Statistical analyses were performed using RStudio software Version 4.2.1 (Posit Software, Boston, MA, USA). Continuous variables are presented as mean ± standard deviation (SD) and were compared between groups using independent samples t-tests. Categorical variables are presented as frequencies and percentages and were compared using chi-square tests or Fisher’s exact test, as appropriate. Odds ratios (OR) with 95% confidence intervals (CI) were calculated to assess the association between Receptiva testing status and anatomical site involvement. A two-sided *p*-value of <0.05 was considered statistically significant. In total, 487 patients presented with infertility as their primary complaint. The study population was intentionally selected to represent patients with unexplained infertility, defined by normal results across standard fertility evaluations. Inclusion criteria required regular menstrual cycles (28–35 days), normal hysterosalpingography (HSG), and normal semen analysis or use of donor sperm. By design, patients with prior evidence of endometriosis were excluded. This approach was chosen to examine the utility of noninvasive testing in the specific clinical scenario where endometriosis is not initially suspected, which represents a key diagnostic challenge in this population. Exclusion criteria included patients with prior visual evidence of endometriosis 134 (e.g., identified via transvaginal ultrasound or MRI), prior laparoscopic or histological confirmation of endometriosis, or evidence of other anatomic abnormalities (e.g., uterine septa). After applying these criteria, the final study population consisted of 195 patients. Importantly, the diagnosis of unexplained infertility was established for all patients prior to diagnostic video-laparoscopy, based on the absence of identifiable causes through standard fertility evaluation. The subsequent finding of endometriosis at surgery thus represented the previously undetected etiology of their infertility.

Patients were divided into two groups: those who had undergone noninvasive (BCL6) diagnostic testing (Receptiva group) and those who had not (No Receptiva group). Patients were grouped by endometriosis stage, and the study examined whether they had previously undergone endometriosis BCL6 noninvasive diagnostic testing. Additionally, the analysis determined the average number of endometriosis locations found laparoscopically within each stage and identified the anatomical sites with the highest prevalence of endometriosis across all patients.

## 3. Results

Data were collected on each patient’s demographic background, pre-evaluation risk assessment for endometriosis, and surgical and histopathological findings. Among the 195 patients with unexplained infertility who underwent diagnostic video-laparoscopy for the evaluation, diagnosis, and treatment of endometriosis or endometrial-like glands and stroma, 43 patients had previously taken a noninvasive Receptiva BCL6 overexpression test (Receptiva group), while 152 had not (No Receptiva group) (see Table 1).

The average age of patients in the Receptiva Positive group was 37.34 ± 3.94 years, compared to 36.00 ± 4.00 years in the No Receptiva group. Both groups had similarly high levels of educational attainment. In the Receptiva group, 23 out of 43 patients (53.49%) had completed a postgraduate degree, and 16 (37.21%) held a bachelor’s degree. In the No Receptiva group, 70 out of 152 patients (46.05%) had completed a postgraduate degree, while 67 (44.08%) had a bachelor’s degree.

As outlined in the inclusion criteria, all patients in both groups (100%) had regular menstrual cycles, a partner with a normal semen analysis or a sperm donor, and no history of endometriosis-related surgery. Among the Receptiva group, 17 out of 43 (39.53%) had previously undergone intrauterine insemination (IUI), 32 (74.42%) had attempted in vitro fertilization (IVF), and 32 (74.42%) had received hormonal therapies. Among No Receptiva group patients, 49 out of 152 (32.24%) had undergone IUI, 66 (43.42%) had attempted IVF, and 87 (57.24%) had received hormonal treatments. Notably, the majority of patients in both groups had exhausted at least one form of assisted reproductive technology (ART) prior to surgical referral.

All patients in this study (100%) were evaluated, diagnosed, and treated for endometriosis through surgical and histopathological confirmation. Among the 43 Receptiva group, 41 (95.3%) tested positive for endometrial BCL6 overexpression, yet all 43 (100%) were histopathologically confirmed to have endometriosis or endometrial-like glands and stroma. Patients who underwent Receptiva testing were found to have the following distribution in the staging of endometriosis: Two (4.65%) patients with Stage I; Thirteen (30.23%) patients with Stage II; Fifteen (34.88%) patients with Stage III; and Thirteen (30.23%) patients with Stage IV. Patients who did not have Receptiva testing were found to have the following distribution: Three (1.97%) patients with Stage I; Forty-two (27.63%) patients with Stage II; Thirty-three (21.71%) patients with Stage III; and Seventy-four (48.68%) patients with Stage IV. A *p*-value of 0.115 was found between the two groups, and a statistically significant *p*-value of 0.038 was found when comparing the patients with stage III and stage IV between the two groups (See Table 2).

Additionally, patients who underwent Receptiva testing had fewer anatomical sites affected by endometriosis (3.14 ± 2.09) compared to those who did not undergo the test (3.93 ± 2.26). Furthermore, a greater proportion of patients in the No Receptiva group were diagnosed with Stage IV endometriosis during surgical evaluation. In both cohorts, endometriosis was most frequently identified in the ureter—affecting 48.84% (21/43) of the Receptiva group and 57.24% (87/152) of the No Receptiva group—followed by the rectovaginal septum, with a prevalence of 41.86% (18/43) and 40.79% (62/152), respectively. Ovarian involvement was also observed; however, data on prevalence, odds ratios, and *p*-values are limited due to the clinical decision to preserve ovarian tissue whenever possible, thereby restricting the frequency of excisional sampling in this region (see Table 3).

## 4. Discussion

The findings of this study further affirm the value of diagnostic video-laparoscopy as a pivotal tool in the diagnosis and management of endometriosis, particularly among patients with unexplained infertility. Our comparison revealed that patients who did not pursue noninvasive diagnostic testing (BCL6) prior to surgical intervention presented with more advanced disease, including a greater number of endometriotic lesions across a wider distribution of anatomical locations. These results highlight an ongoing gap in early identification and intervention, despite advances in biomarker development and risk screening. This persistent gap in endometriosis surveillance and treatment continues to contribute to delayed diagnosis and prolonged patient suffering, and must be urgently addressed to help alleviate the burden experienced by millions of women worldwide.

The association between diagnostic delay and disease burden is well-documented, with prior studies indicating that longer intervals between symptom onset and definitive diagnosis correlate with higher disease severity, greater surgical complexity, and poorer reproductive outcomes. Our findings reinforce this association and illustrate how early clinical suspicion—guided by noninvasive markers such as BCL6 overexpression, miRNA panels, and patient history questionnaires—can facilitate earlier access to definitive care, even if these tools have not yet reached the threshold to replace laparoscopy as the diagnostic gold standard [29,31]. Indeed, multiple studies have validated the clinical utility of BCL6 testing for endometriosis diagnosis. Evans-Hoeker et al. reported a BCL6 sensitivity of 94% and specificity of 75% with a positive predictive value of 96% [33]. The findings established BCL6 as a novel diagnostic marker, demonstrating an area under the curve of 94% for differentiating patients with and without endometriosis [33]. These findings support the use of BCL6 testing as a valuable tool for identifying patients who may benefit from surgical evaluation. In practical terms, integrating ReceptivaDx testing into the clinical workflow could streamline the diagnostic pathway for patients with unexplained infertility. Rather than proceeding directly to empirically assisted reproductive technologies, clinicians could use a positive BCL6 result to justify earlier referral to endometriosis specialists for diagnostic laparoscopy. This approach may reduce the number of failed ART cycles patients undergo before receiving a definitive diagnosis, thereby shortening time-to-treatment and potentially improving reproductive outcomes.

Additionally, by characterizing the most common anatomical sites of endometriotic lesions in our cohort, this study contributes to the surgical community’s efforts to optimize laparoscopic technique and diagnostic yield. Better awareness of high-yield sites for lesion identification, especially in patients with subtle or atypical symptom profiles, can lead to more comprehensive excision, potentially improving fertility outcomes, reducing recurrence, and mitigating long-term risks such as the development of gynecologic cancers and malignancies [5,29,31].

Notably, a substantial proportion of patients in this study had undergone unsuccessful ART before receiving a surgical diagnosis of endometriosis. This trend reflects a concerning pattern in which patients are funneled into costly and often ineffective fertility treatments without first undergoing appropriate evaluation for underlying conditions. However, only a small proportion of patients in our cohort reported undergoing noninvasive biomarker testing prior to surgery. This may be due to response bias or a broader lack of awareness among generalists and primary care providers regarding available diagnostic innovations. These findings echo the conclusions of previous studies and further support the call for reform in medical education to prioritize early recognition and evaluation of chronic, often-overlooked conditions like endometriosis.

Despite promising advances in Health-Tech tools and serum-based diagnostics, current noninvasive approaches still fall short of fully characterizing the severity and distribution of disease, limiting their use as standalone diagnostic tools. Nevertheless, their role in triaging patients for surgical referral should not be underestimated. In the world of Health-Tech, the Free Endometriosis Risk Advisor (EndoRA) App has been shown to score 93.1% sensitivity and 5.9% specificity in detecting endometriosis, with a Positive Predictive Value of 94.1% [26]. Likewise, there is an emergence in Fem-Tech start-ups that are focusing on endometriosis diagnosis and care through the use of mobile apps and wearable technologies [32]. Additionally, there has been a pivotal focus on endometriosis in the field of biotechnology. A recent study published showed that a research group developed a concept design of a testing device that detects HMGB1, which is another protein associated with endometriosis development [34]. 

When thoughtfully implemented, such tools can reduce the diagnostic latency that has long hindered endometriosis care. This is especially critical for patients in underserved populations, where systemic barriers to specialized care are compounded by delays in referral, diagnostic misclassification, and inadequate reproductive health education.

### 4.1. Practical Significance of the Findings

The practical significance of this study lies in its demonstration that noninvasive preoperative diagnosis can meaningfully influence the clinical trajectory of patients with unexplained infertility by facilitating earlier identification of endometriosis at a less advanced stage. Patients who underwent BCL6 testing prior to surgery exhibited a lower disease burden, with significantly fewer involved anatomical sites and a reduced proportion of high-stage disease compared with patients who did not undergo preoperative testing. These findings suggest that incorporating noninvasive screening into infertility evaluation may help clinicians triage patients more effectively for timely surgical referral, rather than delaying diagnosis until advanced disease develops.

From a clinical standpoint, earlier detection of endometriosis has direct implications for surgical planning, fertility preservation, and reproductive outcomes, as lower disease burden is associated with less complex surgery and potentially improved response to subsequent fertility treatments. Our results support the practical integration of noninvasive testing as an adjunct to clinical decision-making, enabling earlier intervention, reducing diagnostic delay, and optimizing care pathways for patients with unexplained infertility suspected of having endometriosis.

### 4.2. Strengths and Limitations

As discussed throughout this article, the findings of this study contribute to a deeper understanding of the future of precision medicine in the diagnosis and treatment of endometriosis. However, one of the most significant limitations of the study is the imbalance between patients who had undergone the ReceptivaDx endometrial BCL6 overexpression test and those who had not. This discrepancy may stem from several factors, including patient self-report bias when providing medical histories, a lack of understanding about what the ReceptivaDx test entails—leading to confusion about whether they had received it—and limited utilization of the test by referring physicians who may be unaware of noninvasive diagnostic options. Additionally, there are potential selection biases, raising questions about whether the study population accurately represents the broader global population of endometriosis patients. This concern is especially relevant given the known barriers to accessing specialized care, which may disproportionately affect individuals from diverse socioeconomic or geographic backgrounds. Furthermore, the single-center, retrospective design of this study may limit the generalizability of findings to other clinical settings or patient populations. The unequal sample sizes between groups (43 Receptiva vs. 152 No Receptiva) reflect real-world clinical practice where noninvasive biomarker testing is not yet routinely offered to all patients with unexplained infertility. While this may have limited statistical power to detect smaller effect sizes, the comparison remains valuable for evaluating whether patients who undergo preoperative screening present with different disease characteristics. Multi-center prospective studies with more balanced cohorts are needed to validate these results.

### 4.3. Future Direction

Future research should focus on prospective, multi-center studies to validate the predictive value of BCL6 testing across diverse patient populations, and longitudinal studies are needed to determine whether integrating noninvasive testing into infertility algorithms improves long-term fertility outcomes and patient quality of life. Additionally, future research should evaluate the cost-effectiveness of incorporating noninvasive biomarker testing into standard infertility evaluation protocols. Such analyses would help determine whether routine BCL6 testing reduces overall healthcare expenditures by decreasing unsuccessful ART cycles and expediting appropriate surgical intervention.

## 5. Conclusions

In this study of 195 patients with unexplained infertility and confirmed endometriosis, those who underwent preoperative Receptiva BCL6 testing had significantly fewer affected anatomical sites and a lower proportion of high-stage disease compared to those who did not undergo testing. These findings suggest that noninvasive biomarker testing may facilitate earlier diagnosis and intervention, before disease progression. While surgical visualization and histological confirmation remain the diagnostic gold standard, integrating BCL6 testing into the clinical workflow for patients with unexplained infertility may help reduce diagnostic delays and improve patient outcomes. Future prospective, multi-center studies with balanced cohorts are needed to validate these findings across diverse populations and evaluate the cost-effectiveness of incorporating noninvasive testing into standard infertility evaluation protocols. Only through such a comprehensive approach can we hope to mitigate the clinical, emotional, and economic burdens of this often-invisible disease.

## Figures and Tables

**Table 1:** Baseline characteristics of the study population.

	Receptiva Group	No Receptiva Group	*p*-Value
	N = 43	N = 152	
Age (mean ± SD)	37.34 ± 3.94	36.00 ± 4.00	0.0500
BMI (mean ± SD)	23.6 ± 3.96	23.33 ± 3.57	0.630
Education (N (%))			0.592
Postgraduate	23 (53.49%)	70 (46.05%)	
Bachelor’s	16 (37.21%)	67 (44.08%)	
High School	4 (9.30%)	10 (6.58%)	
No Data	0 (0.00%)	5 (3.29%)	
Receptiva Positive (N (%))	41 (95.3%)	-	-
Normal Menstrual Cycles (N (%))	43 (100%)	152 (100%)	1.00
Normal Semen Analysis (N (%))	43 (100%)	152 (100%)	1.00
Previously Had Endometriosis Surgery (N (%))	0 (0%)	0 (0%)	1.00
Previous ART (N (%))			0.546
IUI	17 (39.53%)	49 (32.24%)	
IVF	32 (74.42%)	66 (43.42%)	
Hormonal Treatments	32 (74.42%)	87 (57.24%)	

BMI: Body Mass Index; ART: Assisted Reproductive Techniques; IUI: Intrauterine Insemination; IVF: In Vitro Fertilization. Receptiva Group = All patients who have taken a noninvasive Receptiva BCL6 overexpression test (only 95.3% had a positive test result). No Receptiva Group = Patients who did not take the noninvasive Receptiva BCL6 overexpression test.

**Table 2:** Comparison of surgical findings between patients who underwent noninvasive diagnostic testing and patients who did not before seeking video-laparoscopic diagnosis and treatment for endometriosis.

	Receptiva Group	No Receptiva Group	*p*-Value
	N = 43	N = 152	
Endometriosis Confirmed in Surgery (N (%))	43 (100%)	152 (100%)	1.00
Endometriosis Confirmed in Histopathology Exam (N (%))	43 (100%)	152 (100%)	1.00
Endometriosis Stage (N (%))			0.115
I	2 (4.65%)	3 (1.97%)	
II	13 (30.23%)	42 (27.63%)	
III	15 (34.88%)	33 (21.71%)	
IV	13 (30.23%)	74 (48.68%)	
High-Stage Endometriosis (III and IV) (N (%))	28 (65.12%)	107 (70.39%)	0.038
Number of involved anatomical locations (mean ± SD)	3.14 ± 2.09	3.93 ± 2.26	0.04

Note: Receptiva Group = All patients who have taken a noninvasive Receptiva BCL6 overexpression test. No Receptiva Group = Patients who did not take the noninvasive Receptiva BCL6 overexpression test.

**Table 3:** Comparison of the distribution of anatomical involvement of endometriosis between patients who underwent noninvasive diagnostic testing and patients who did not before seeking video-laparoscopic diagnosis and treatment for endometriosis.

Anatomical Site	Receptiva Group	No Receptiva Group	*p*-Value	OR (95% CI)
Ureter (N (%))	21 (48.84%)	87 (57.24%)	0.33	0.71 (0.36, 1.41)
Rectum (N (%))	4 (9.30%)	30 (19.74%)	0.11	0.42 (0.14, 1.26)
Bladder (N (%))	7 (16.28%)	37 (24.34%)	0.27	0.60 (0.25, 1.47)
Parametrium (N (%))	9 (20.93%)	40 (26.32%)	0.47	0.74 (0.33, 1.68)
Rectosigmoid Colon (N (%))	4 (9.30%)	23 (15.13%)	0.33	0.57 (0.19, 0.76)
Pararectal Right (N (%))	7 (16.28%)	43 (28.29%)	0.11	0.49 (0.20, 1.19)
Pararectal Left (N (%))	6 (13.95%)	39 (25.66%)	0.11	0.47 (0.18, 1.20)
Rectal Bulb (N (%))	1 (2.33%)	13 (8.55%)	0.16	0.25 (0.03, 2.00)
Utero-Sacral ligament (N (%))	2 (4.65%)	6 (3.95%)	0.84	1.19 (0.23, 6.10)
Recto-Vaginal Septum (N (%))	18 (41.86%)	62 (40.79%)	0.90	1.04 (0.52, 2.07)
Ovaries (N (%))	6 (13.95%)	39 (25.66%)	0.11	0.47 (0.18, 1.20)
Fallopian Tubes (N (%))	4 (9.30%)	6 (3.95%)	0.16	2.49 (0.67, 9.28)
Uretro-Vesical Junction Right (N (%))	10 (23.26%)	29 (19.08%)	0.50	1.28 (0.57, 2.90)
Uretro-Vesical Junction Left (N (%))	14 (32.56%)	36 (23.68%)	0.24	1.55 (0.74, 3.26)
Anterior Cul-de-sac (N (%))	4 (9.30%)	10 (6.58%)	0.54	1.45 (0.43, 4.90)
Posterior Cul-de-sac (N (%))	2 (4.65%)	10 (6.58%)	0.64	0.69 (0.146, 3.29)
Pelvic Sidewall (N (%))	11 (25.58%)	52 (34.21%)	0.29	0.66 (0.31, 1.42)
Diaphragm (N (%))	5 (11.63%)	35 (23.03%)	0.10	0.44 (0.161, 1.20)

Note: Ovarian involvement data are limited due to the clinical decision to preserve ovarian tissue whenever possible, thereby restricting the frequency of excisional sampling in this region. Receptiva Group = All patients who have taken a noninvasive Receptiva BCL6 overexpression test. No Receptiva Group = Patients who did not take the noninvasive Receptiva BCL6 overexpression test.

## Data Availability

Data will be made available to the editors of the journal for review or query upon request.

## Abbreviations

The following abbreviations are used in this manuscript:

ART	Assisted Reproductive Technology
BCL6	B-cell Lymphoma 6 Protein
BMI	Body Mass Index
HSG	Hysterosalpingography
HMGB1	High Mobility Group Box 1 Protein
ICAM-1	Intercellular Adhesion Molecule-1
IUI	Intrauterine Insemination
IVF	In Vitro Fertilization
miRNA	MicroRNA
MRI	Magnetic Resonance Imaging
SD	Standard Deviation
